# Chronic administration of *Angelica sinensis* polysaccharide effectively improves fatty liver and glucose homeostasis in high-fat diet-fed mice

**DOI:** 10.1038/srep26229

**Published:** 2016-05-18

**Authors:** Kaiping Wang, Peng Cao, Hanxiang Wang, Zhuohong Tang, Na Wang, Jinglin Wang, Yu Zhang

**Affiliations:** 1 Hubei Key Laboratory of Natural Medicinal Chemistry and Resource Evaluation, Tongji Medical College of Huazhong University of Science and Technology, 430030, Wuhan, China; 2 Department of Pharmacy, Renmin Hospital of Wuhan University, No. 99, Zhangzhidong Road, 430060, Wuhan, China; 3 Union Hospital of Huazhong University of Science and Technology, Department of Pharmacy, No. 1227, Jiefang Road, 430030, Wuhan, China

## Abstract

This study aimed to investigate the therapeutic effects of *Angelica sinensis* polysaccharide (ASP), an active component derived from a water extract of *Angelica sinensis*, in high-fat diet (HFD)-fed BALB/c mice. The potential mechanisms underlying the activity of this compound were also considered. Specifically, serum and hepatic biochemical parameters were evaluated, and key proteins involved in the lipid/glucose metabolism were analyzed. Long-term feeding with a HFD induced severe fatty liver and hyperglycemia. Histological examination clearly showed that ASP reduced lipid accumulation in the liver and attenuated hepatic steatosis in HFD-fed mice. In addition, ASP markedly alleviated serum and liver lipid disorders and fatty liver via the upregulation of PPARγ expression and the activation of adiponectin-SIRT1-AMPK signaling. Furthermore, ASP also significantly relieved severe oxidative stress, demonstrating that ASP might attenuate nonalcoholic fatty liver disease via a “two-hit” mechanism. In addition, ASP reduced blood glucose levels and ameliorated insulin resistance via the regulation of related metabolic enzymes and by activating the PI3K/Akt pathway in HFD-fed mice. Our findings revealed that ASP might be used as an alternative dietary supplement or health care product to ameliorate metabolic syndrome in populations that consistently consume HFDs.

Environmental factors, such as a high-fat diet (HFD) and a sedentary lifestyle, are the causes of the current epidemic of metabolic syndrome, which is characterized by a group of metabolic risks including hyperglycemia, hyperlipidemia, hypertension and insulin resistance^1^. Excessive exposure to a HFD is believed to play a key role in the pathogenesis of metabolic damages and has led to an increased number of individuals with nonalcoholic fatty liver disease (NAFLD) and diabetes^2^,^3^. NAFLD, which represents a spectrum of liver pathologies that range from simple steatosis to severe hepatitis, fibrosis, cirrhosis, and hepatocellular carcinoma, has become the most common cause of chronic liver diseases^4^. HFD-induced hyperglycemia increases the risk of development of type 2 diabetes, which is also an epidemic disease in modern society. Changes in dietary habits merit particular consideration because a HFD leads to severe hyperglycemia, metabolic damages and fatty liver. However, the modification of dietary habits and lifestyles are not readily accepted in many developed countries. Therefore, dietary supplementation or consumption of health foods could be an alternative means for the prevention of metabolic syndrome.

Traditional Chinese medicines are rich sources of bioactive substances that can be used to pretreat or cure various types of human diseases^5^. Polysaccharides are polymeric carbohydrate macromolecules composed of long chains of monosaccharide units that are connected by various glycosidic linkages. Currently, an increasing number of studies have focused on polysaccharides extracted from different Chinese medicines due to their potential pharmacological activities. Many types of polysaccharides have shown appreciable effects in the amelioration of NAFLD or diabetes, such as *Lycium barbarum* polysaccharide^6^, Radix Hedysari polysaccharide^7^, *Aureobasidium pullulans*-derived β-glucan^8^, *Rehmannia glutinosa* polysaccharide^9^ and *Ganoderma lucidum* polysaccharide^10^. Specifically, these polysaccharides have demonstrated hypolipidemic/hypoglycemic effects or antioxidative properties. *Angelica sinensis* polysaccharide (ASP), a biomacromolecule isolated from the roots of *Angelica sinensis*, has drawn accumulating attention recent years for its various bioactivities, such as hematopoietic, immunomodulatory, hepatoprotective, and antioxidant activities, among others^11^,^12^,^13^,^14^. In addition, our group has demonstrated that ASP has hypoglycemic and hypolipidemic effects in a mouse model of type 2 diabetic mice and that this action was mediated by an elevation of glycogen levels and a reduction of inflammatory factors^15^.

In the current study, we established an HFD-induced mouse model that resulted in fatty liver, hyperglycemia, as well as severe oxidative stress^16^,^17^. We then explored the potential effects of ASP on the improvement of metabolic dysfunction and oxidative stress in HFD-fed mice by examining various physiological parameters and investigating the mechanisms underlying these beneficial properties. Our findings demonstrate that ASP is an excellent candidate for the prevention of HFD-induced metabolic syndrome. Therefore, to prevent the NAFLD or diabetes, ASP can be used as a health care product or food supplement in populations that consistently consume HFDs.

## Results

### ASP does not affect body weight or liver weight in HFD-fed mice

**Figure 1:**
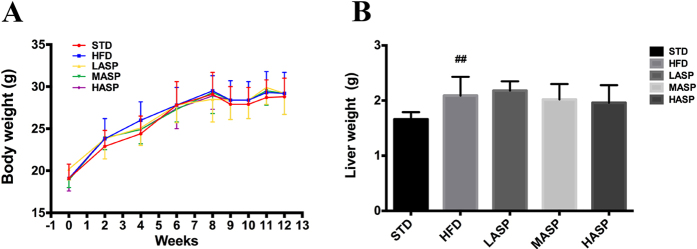
Body weight (**A**) and liver weight (**B**) in STD-fed and HFD-fed mice. The values are given as the mean ± S.D. (n = 12). ^#^*P* < 0.05, ^##^*P* < 0.01 versus the STD group. *P < 0.05, **P < 0.01 versus the HFD group.

Mice were fed a HFD for 12 weeks and then orally administered either a low-dose, middle-dose or high-dose of ASP (80, 160 and 320 mg·kg^−1^ per day, referred as LASP, MASP and HASP group, respectively) over the last 4 weeks. Interestingly, body weight was not significantly different between the control HFD-fed mice and the mice fed a standard diet (STD) during the experimental process. In addition, ASP intervention in the final 4 weeks did not affect body weight (Fig. 1A). In addition, the weight of the livers were measured after the mice were euthanized. In contrast to body weight, liver weight was significantly increased in HFD-fed mice (2.09 ± 0.34 g) in comparison with the STD-fed mice (1.66 ± 0.13 g). Middle-dose and high-dose ASP treatment slightly lowered the liver weight of HFD-fed mice (2.02 ± 0.28 g and 1.96 ± 0.32 g, respectively), but this effect failed to reach statistical significance (Fig. 1B).

### ASP reduces hepatic steatosis, ameliorates fatty liver and attenuates liver injury in HFD-fed mice

**Figure 2:**
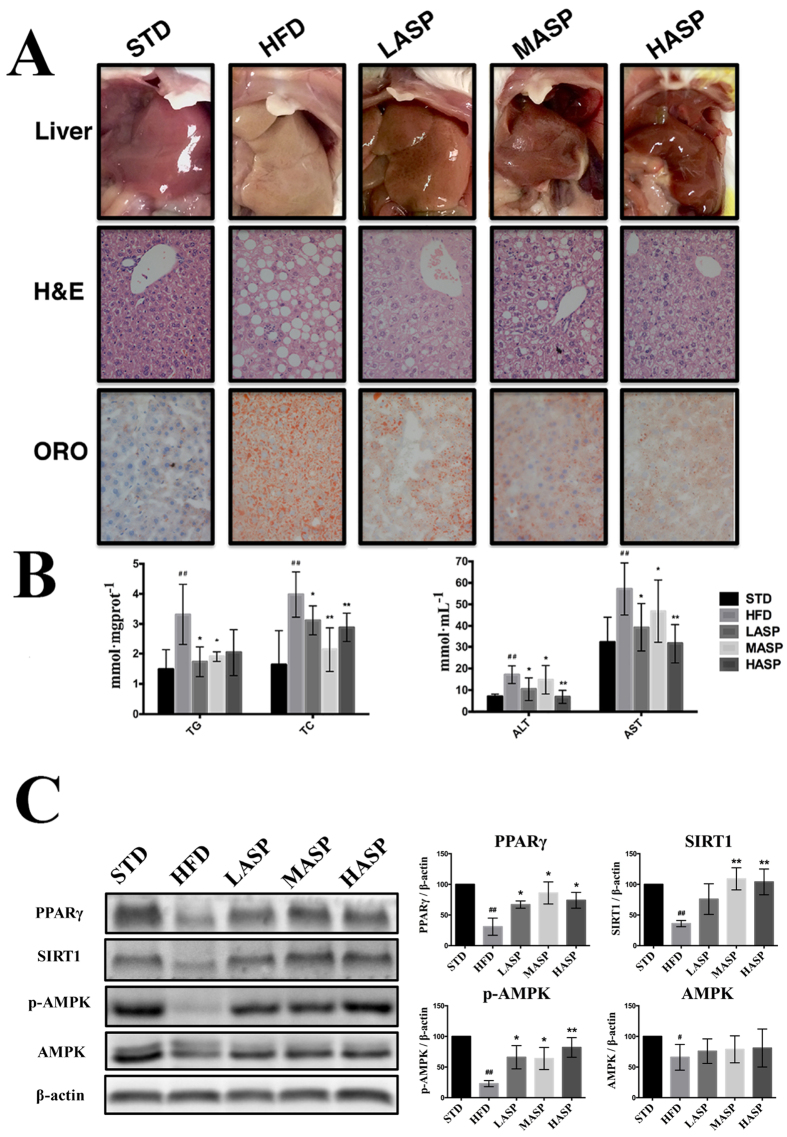
The appearance and histological assessment (400x magnification) of the livers (**A**), hepatic TC/TG and serum ALT/AST determination (**B**) and representative immunoblots of the protein expression levels of PPARγ, SIRT1, p-AMPK and AMPK in the livers (**C**) of STD-fed and HFD-fed mice. Full-length blots are presented in Supplementary Figure 1. The values are given as the mean ± S.D. (n = 12 or 3). ^#^*P* < 0.05, ^##^*P* < 0.01 versus the STD group. *P < 0.05, **P < 0.01 versus the HFD group.

Initially, we observed pale and dull livers in mice fed a HFD but bright red and glossy-appearing livers in the other groups (Fig. 2A). To examine histology of these livers, we performed hematoxylin and eosin (H&E) and Oil Red O (ORO) staining. The H&E-stained tissue slices showed that the livers of mice fed a STD had orderly, well-arranged and distinct cell borders, hepatic cords, and round central nuclei. However, we observed hepatocyte hypertrophy and severe hepatic steatosis in mice fed an HFD, and effect that was markedly alleviated after the ASP intervention. Tissue sections stained with ORO clearly revealed that HFD consumption resulted in increased lipid content in the liver. Likewise, ASP treatment led to a significant amelioration of lipid accumulation in the HFD-fed mice.

To further analyze the effects of ASP on the lipid profiles of the treated mice, we measured total cholesterol (TC), triglyceride (TG), low-density lipoprotein cholesterol (LDL-C) and high-density lipoprotein cholesterol (HDL-C) in serum (Table 1) along with TC and TG in the liver (Fig. 2B). The HFD-fed mice exhibited significant dyslipidemia when compared with those fed a STD. The administration of ASP alleviated all of the lipidic biomarkers, and high-dose ASP reversed the serum TG, TC, HDL-C and LDL-C to nearly normal levels in comparison with the HFD-fed mice. In addition, it was observed that TG and TC levels were significantly elevated in the liver of HFD-fed mice by 1.2- and 1.4-fold, respectively, compared with STD-fed mice. In accordance with the role of ASP on serum lipid profiles, ASP significantly ameliorated hepatic lipid abnormality in HFD-fed mice. ASP also significantly reversed the elevated levels of alanine aminotransferase (ALT) and alanine aminotransferase (AST) (Fig. 2B), which were the indicators of liver injury.

### ASP ameliorates fatty liver by upregulating PPARγ expression and activation of adiponectin-SIRT1-AMPK signaling

To understand the potential mechanism of the observed effects, we evaluated signaling pathways and key that have been proteins implicated in lipid metabolism modulation. Western blot analysis showed that PPARγ expression was markedly diminished after HFD treatment. However, as shown in Fig. 2C, western blots revealed that PPARγ expression was significantly increased by all doses of ASP. In agreement with the result that reduced serum adiponectin was partially reversed by ASP intervention (Table 1), SIRT1-AMPK signaling was also significantly activated to a certain extent, as evidenced by the elevation of SIRT1 and p-AMPK expression after ASP treatment (Fig. 2C).

### ASP attenuates hepatic oxidative stress and enhances antioxidant enzyme activity in HFD-mice

**Figure 3:**
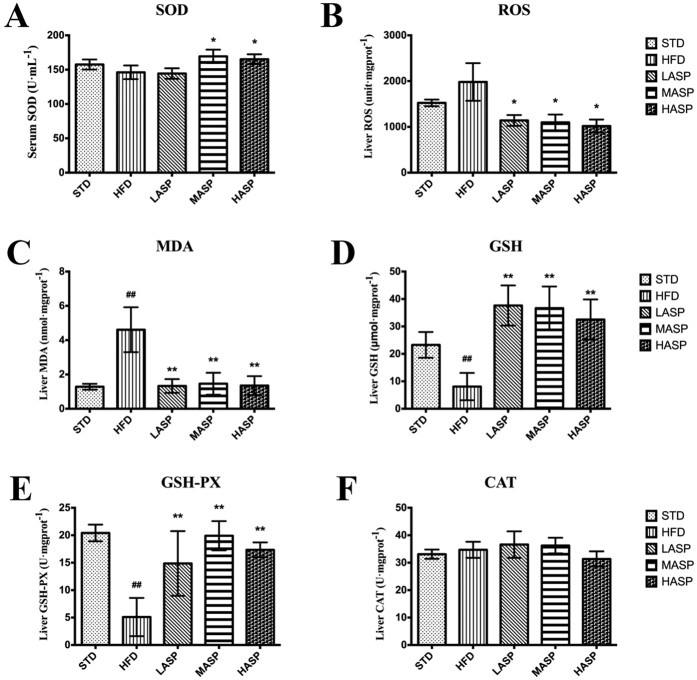
Serum SOD (**A**), hepatic ROS (**B**), MDA (**C**), GSH (**D**), GSH-PX (**E**) and CAT (**F**) levels or activities of STD-fed and HFD-fed mice. The values are given as the mean ± S.D. (n = 12). ^#^*P* < 0.05, ^##^*P* < 0.01 versus the STD group. *P < 0.05, **P < 0.01 versus the HFD group.

The “two-hit” mechanism suggests that the development of NAFLD is influenced by both hepatic steatosis and oxidative stress. Thus, we evaluated the oxidative stress and antioxidant systems. Serum superoxide dismutase (SOD) levels were markedly elevated in the middle- and high-dose ASP-treated mice (Fig. 3A) compared with the HFD-fed mice. Figure 3B,C shows the liver concentrations of reactive oxygen species (ROS) and malondialdehyde (MDA), which are commonly employed indicators of oxidative stress. Abnormal hepatic ROS levels were significantly decreased by low-, middle- and high-dose ASP interventions (decreases of 42.5%, 44.7% and 48.7%, respectively) compared with the HFD-fed mice. Similarly, MDA levels were increased by 2.6-fold in HFD-fed mice compared with the STD-fed mice. Interestingly, treatment with ASP for 4 weeks at all doses resulted in complete recovery of the abnormal MDA levels. In addition, glutathione (GSH) and glutathione peroxidase (GPx) activities were significantly decreased by 65.3% and 75.0%, respectively, after a HFD treatment for 12 weeks when compared with the STD-fed mice (Fig. 3D,E). However, ASP resulted in a significant recovery of the impaired levels of these markers in the liver. Furthermore, no statistically significant differences were observed with respect to catalase (CAT) activity between the groups, although a high-dose ASP slightly diminished CAT levels (Fig. 3F).

### ASP alleviates hyperglycemia and insulin resistance in HFD-fed mice

As expected, a HFD induced marked elevations of both Hemoglobin A1c (HbA1c) and fasting blood glucose (FBG) (Table 1). Nevertheless, the low-, middle- and high-dose ASP treatments significantly reduced HbA1c levels by 26.6%, 24.9% and 28.6%, respectively, compared with HFD-fed mice. Consistent with the effect of ASP on HbA1c, glucose levels were diminished by 33.9%, 35.7% and 41.1% after daily administration of 80, 160 and 320 mg·kg^−1^ ASP, respectively. Intriguingly, the serum insulin level of HFD-fed mice was not significantly different from that of STD-fed mice (Table 1). The middle dose of ASP slightly elevated the insulin concentration (*P* = 0.045) compared with the HFD-fed mice, but neither the low- nor the high-dose ASP treatment caused any marked fluctuations on insulin levels.

To explore the effect of ASP on insulin resistance, we determined the homeostasis model assessment-insulin resistance (HOMA-IR) index, as described in the Methods. It was demonstrated that the HFD-fed mice manifested marked insulin resistance. Specifically, the HOMA-IR index of HFD-fed animals was significantly higher than that of STD-fed mice. However, as determined by the HOMA-IR index, low- and high-dose ASP treatment significantly alleviated insulin resistance in comparison with the HFD-fed mice (approximately 31.7% and 33.3% reduction, respectively). In addition, abnormal resistin concentrations in the serum were markedly reversed by the high-dose ASP treatment (Table 1).

### ASP improves glucose tolerance in HFD-fed mice and enhances insulin response in insulin resistant HepG2 cells

**Figure 4:**
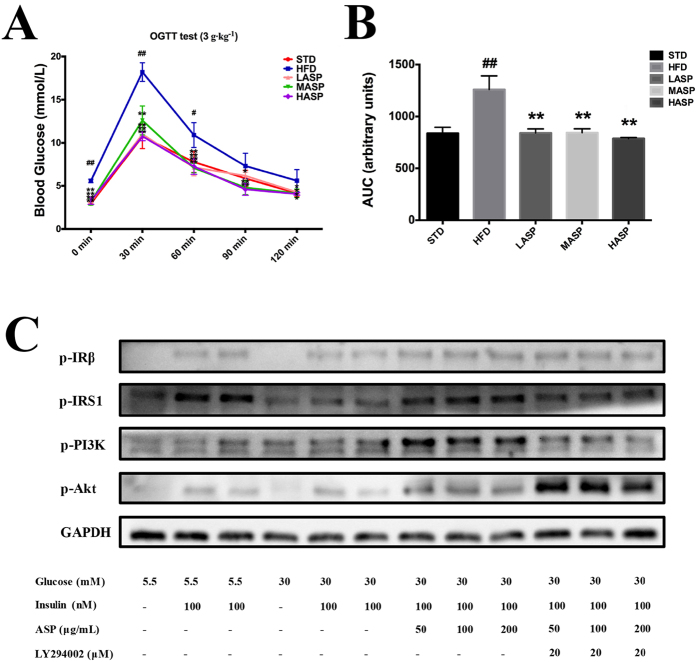
Time course changes in the levels of blood glucose during OGTT (**A**) and area under the curve for glucose concentrations calculated using the trapezoidal rule during the OGTT test (**B**) in STD-fed and HFD-fed mice. The values are given as the mean ± S.D. (n = 12). ^#^*P* < 0.05, ^##^*P* < 0.01 versus the STD group. *P < 0.05, **P < 0.01 versus the HFD group. Insulin response test in HepG2 cells (**C**). Full-length blots are presented in Supplementary Figure 2.

The effect of ASP on oral glucose tolerance test (OGTT) is presented in Fig. 4A,B. The glucose tolerance capacity of the mice fed a HFD was severely impaired when compared with that of STD-fed mice. The blood glucose levels of the HFD-fed mice were significantly higher than those of the STD-fed mice. However, there were no marked differences between the glucose levels of STD-fed mice and ASP-treated mice based on OGTT monitoring. Compared to the large area under the curve (AUC) of HFD-fed mice, the AUCs of STD-fed mice and ASP-treated mice were significantly smaller.

In addition, we tested the insulin response on insulin-resistant HepG2 cells, and our results showed that ASP significantly enhanced insulin signaling after 100 nM insulin stimulation for 10 min. The expression of levels key proteins, including p-IRβ, p-IRS1, p-PI3K and p-Akt, were greatly increased in ASP-treated cells compared with model control cells. Moreover, the PI3K inhibitor LY294002 offset the effect of ASP, confirming that ASP improved insulin sensitivity.

### ASP regulates hepatic glycogen content and glucose metabolism enzyme levels in HFD-fed mice

**Figure 5:**
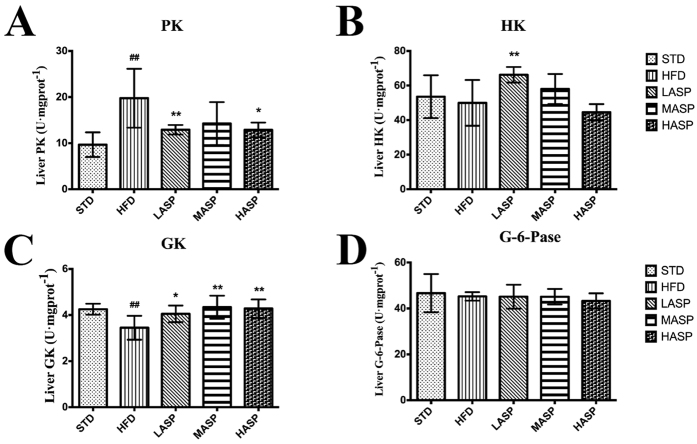
Hepatic PK (**A**), HK (**B**), GK (**C**), and G-6-Pase (**D**) levels. The values are given as the mean ± S.D. (n = 12). ^#^*P* < 0.05, ^##^*P* < 0.01 versus the STD group. *P < 0.05, **P < 0.01 versus the HFD group.

We first examined the content of hepatic glycogen in each group (Table 1). In HFD-fed mice, the hepatic glycogen concentration was reduced by 72.5% when compared with STD-fed mice. The low, middle, and high doses of ASP significantly increased the glycogen levels by 2.0-, 1.6- and 2.1-fold, respectively, in comparison with the STD-fed mice. To evaluate the hepatic enzymes that are involved in glucose metabolism, we next determined the enzymatic activity of pyruvate kinase (PK) and hexokinase (HK) as well as the expression of glucokinase (GK) and glucose-6-phosphatase (G-6-Pase) in the liver. Figure 5A,C shows that the HFD treatment increased PK activity by 104.5% and decreased GK expression by 18.8% compared with STD-fed mice. ASP therefore restored the impaired levels of PK and GK caused by a HFD. However, the levels of G-6-Pase and HK were not changed after a 12-week HFD or by ASP treatment, except that a low-dose ASP significantly elevated HK activity in HFD-fed mice (Fig. 5B).

### ASP ameliorates insulin resistance via improved insulin signaling in HFD-fed mice

**Figure 6:**
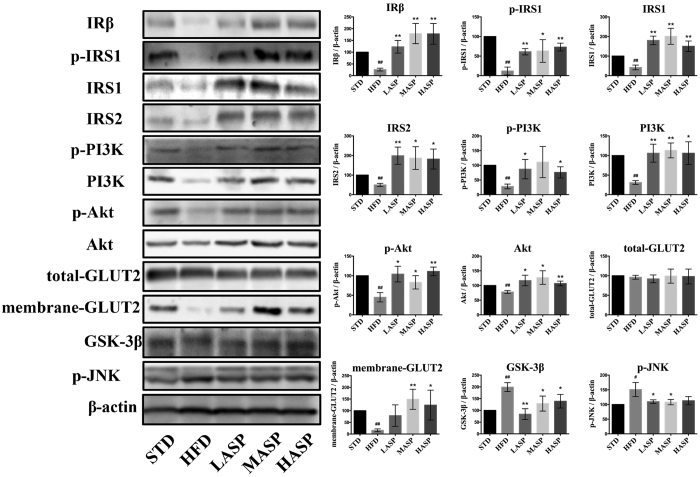
Representative immunoblots of the protein expression levels of InR-β, p-IRS1, IRS-1/2, p-PI3K, PI3K, p-Akt, Akt, total GLUT2, membrane GLUT2, GSK-3β and p-JNK in the livers of STD-fed and HFD-fed mice. Full-length blots are presented in Supplementary Figure 3. The values are given as the mean ± S.D. (n = 3). ^#^*P* < 0.05, ^##^*P* < 0.01 versus the STD group. *P < 0.05, **P < 0.01 versus the HFD group.

To elucidate the potential mechanism underlying the observed ASP-mediated improvement in hyperglycemia and insulin resistance, we evaluated intracellular insulin signaling pathway molecules in the liver. Representative western blot analysis of Insulin Receptor-β (InR-β), phospho-Insulin Receptor Substrate-1 (p-IRS1), Insulin Receptor Substrate-1/2 (IRS1/2), phospho-phosphoinositide-3-kinase (p-PI3K), phosphoinositide-3-kinase (PI3K), phospho-Akt (p-Akt), Akt, total glucose transporter 2 (GLUT2), membrane GLUT2, GSK-3β and phospho-JNK are displayed in Fig. 6. The data clearly show that the expression of InR-β, p-IRS1, IRS1/2, p-PI3K, PI3K, p-Akt, Akt and membrane GLUT2 were significantly decreased in HFD-fed mice compared with STD-fed mice, while GSK-3β was markedly increased. The ASP intervention resulted in a marked restoration in protein expression of these markers. No difference was observed in hepatic total GLUT2 expression between the different groups, while phospho-JNK levels were increased in HFD-fed mice in comparison with STD-fed mice. ASP treatment led to a significant reduction in the expression of phospho-JNK compared with HFD-fed mice.

### ASP does not affect serum hepcidin or TNF-α levels in HFD-fed mice

Apart from parameters associated with glucose and lipid metabolism, we also analyzed the serum levels of hepcidin, a key regulator of iron metabolism in mammals, and inflammatory factor TNF-α. No significant variations in these molecules were observed in any of the groups (Table 1).

## Discussion

ASP is a galactose-rich polysaccharide, and we have demonstrated that it strongly targets the liver via the asialoglycoprotein receptors on the surface of hepatocytes (unpublished data). Considering that the liver is an important organ in the regulation of lipids and glucose metabolism and that ASP is a liver-targeting natural product, we speculated that ASP might have beneficial effects on metabolic diseases. Several studies have demonstrated that ASP and *Angelica sinensis* exerts appreciable hypoglycemic and hypolipidemic effects in diabetic rats^14^,^18^. Based on these studies, we evaluated the beneficial effects of ASP in a long-term HFD treatment-induced mouse model of fatty liver, hyperglycemia, hyperlipidemia, insulin resistance and oxidative stress. Researchers commonly use a HFD to generate valid rodent models with metabolic disorders^19^. In general, the C57BL/6J strain is the most commonly used in obesity research due to their obesity-prone characteristics^20^. However, we chose BALB/c mice in the current study because they manifest more severe hepatic lipid accumulation and vacuolation of hepatocytes^21^, higher serum lipid levels, and elevated blood glucose levels after HFD treatment. In contrast, body weight in this strain is not as variable as that of C57BL/6J mice^22^,^23^. Thus, we established a BALB/c mouse model with severe hepatic defects and explored the effects of ASP treatment on NAFLD and hyperglycemia.

In accordance with previous reports, the body weight of BALB/c mice was not affected by HFD treatment. However, liver weight was significantly increased after a 12-week HFD treatment due to hepatic lipid accumulation and the development of fatty liver. This result was confirmed by histological liver slices and the gross appearance of the liver (Fig. 2). Although ASP failed to reverse the abnormal liver weight, histological examination revealed that ASP improved hepatic steatosis, lipid accumulation and fatty liver to a large degree, and this result was confirmed by the considerable amelioration of lipid profiles in both the serum and liver.

To explore the potential mechanism of the hypolipidemic properties of ASP, we examined adiponectin-SIRT1-AMPK signaling and PPARγ expression in the liver. Adiponectin is a protein that is primarily secreted from adipose tissue and subsequently circulates in the serum. In addition, this protein has been demonstrated to reduce fat accumulation and to increase fatty acid oxidation in the liver^24^. The SIRT1-AMPK axis has emerged as a pivotal signaling system in the lipid-lowering action of adiponectin and in the regulation of adiponectin signaling^25^. Moreover, AMPK and SIRT1, which are two vital metabolic sensors, are closely related to lipid metabolism, and the activation of SIRT1-AMPK signaling in the liver has been found to repress lipogenesis and to increase the rates of fatty acid oxidation^26^,^27^. In this study, we initially found that reduced adiponectin levels were partially restored after ASP treatment, demonstrating that the amelioration of fatty liver might be correlated with adiponectin-mediated signaling. We therefore evaluated adiponectin-SIRT1-AMPK signaling and found that p-AMPK and SIRT1 expressions in the liver were significantly upregulated. Collectively, our results indicated that the alleviation of lipid metabolism disorder and fatty liver in HFD-fed mice might be associated with the regulation of adiponectin-SIRT1-AMPK signaling. In addition, we determined the liver levels of PPARγ, which is a transcription factor involved in the regulation of lipid metabolism and is associated with hepatic lipid accumulation in fatty liver^28^. Our study showed that ASP increased but did not normalize the impaired PPARγ levels, suggesting that the beneficial effect of ASP on lipid metabolism was due, at least in part, to the regulation of PPARγ.

The pathogenesis of NAFLD is usually explained by the “two-hit” theory^29^. The first hit is hepatic steatosis, which is characterized by excess lipid accumulation in the liver, and the second hit is oxidative stress, i.e., the increased generation of reactive oxygen species and enhanced lipid peroxidation. In the present study, long-term feeding with a HFD resulted in severe oxidative stress, as evidenced by reduced concentrations of MDA and ROS, which are common biomarkers of oxidative damage. The endogenous anti-oxidative defense system is usually assessed by the status of enzymatic antioxidants, such as SOD, CAT, GSH and GPx, which play pivotal roles in the defense against oxidative damage. SOD catalyzes the conversion of the superoxide anion radical into peroxides, while CAT is an enzymatic antioxidant that catalyzes the reduction of peroxides^30^. Moreover, GPx converts peroxides into water^31^, whereas reduced GSH provides reducing equivalents for GPx catalyzed reduction^32^. It was clearly observed that a HFD impaired the antioxidant defense system, as evidenced by the suppressed GSH and GPx levels. However, ASP showed considerably improved antioxidant activity; specifically, hepatic GSH and GPx and serum SOD levels were significantly increased after ASP administration. However, in agreement with the previously reported findings^33^, neither a HFD nor ASP affected CAT activity in the liver. Taken together, consistent with other studies showing that ASP improves the antioxidant system in other animal models^11^,^34^, our findings demonstrated that the effects of ASP against NAFLD could be mediated by inhibiting oxidative stress. However, a further in-depth study of the anti-oxidative effect was required to elucidate the mechanism of action.

Glucose metabolism impairment and insulin resistance are found in most NAFLD patients. Indeed, a 12-week HFD treatment also induced glucose metabolism disorder in BALB/c mice. This effect manifested as hyperglycemia, increased blood HbA1c levels, impaired glucose tolerance and decreased hepatic glycogen content. Nevertheless, ASP effectively alleviated all of the parameters implicated in glucose metabolism to levels comparable to STD-fed mice.

To investigate the underlying mechanism for the improvement of hyperglycemia, we examined various liver enzymes that are involved in glucose metabolism. It is well known that blocked glycogenesis and reduced glycolysis, which are the fundamental physiological effects that stimulate gluconeogenesis, are involved in certain metabolic diseases^35^. GK, G-6-Pase, PK and HK are key liver enzymes that are involved in glycolysis and gluconeogenesis. GK phosphorylates glucose to glucose-6-phosphate, while G-6-Pase catalyzes glucose-6-phosphate to free glucose via dephosphorylation^36^. As a glycolytic enzyme, PK is ubiquitously expressed in the liver and converts phosphoenol pyruvate to pyruvate^37^. HK is an enzyme that phosphorylates hexoses, forming hexose phosphate. In most organisms, glucose is the most important substrate of HK, and glucose-6-phosphate acts as the most important product. Our study demonstrated that ASP significantly restored the glycogenic capacity in HFD-fed mice. This beneficial effect might be due, at least in part, to the elevation of GK expression and a reduction of PK activity. Moreover, the fact that a low-dose ASP significantly increased HK activity in HFD-fed mice suggested that the restoration of HK might also contribute to the metabolic process. However, neither a HFD nor ASP affected the expression of G-6-Pase. The detailed relationship between the recovery of FBG and glucose metabolism enzymes remains to be fully explored.

In addition to hyperglycemia and hyperlipidemia, insulin resistance, which is calculated according to the overall levels of blood glucose and serum insulin, is also a pivotal symptom in NAFLD. In general, the serum insulin concentration in HFD-fed C57BL/6J mice was higher than in STD-fed mice due to reduced insulin sensitivity and compensatory hyperinsulinemia. However, an interesting finding in the present study was that a HFD did not significantly affect serum insulin, a result that is consistent with a previous study in which BALB/c mice exhibited protection from HFD-induced hyperinsulinemia^38^. Hyperinsulinemia was not observed in HFD-fed mice, and ASP did not affect serum insulin levels. However, *in vitro* experiments confirmed that ASP could improve insulin sensitivity, exerting a hypoglycemic effect when insulin levels remain unchanged. In addition, the HOMA-IR index of HFD-fed mice was markedly lowed by ASP due to its significant hypoglycemic effect. It is worth noting that a previous study reported BALB/c mice to be resistant to other detrimental effects of HFD, including hyperglycemia and impaired glucose tolerance; these previous results are inconsistent with our findings. The discrepancies might be explained by the different ingredients of the HFD fed to the animals, which consisted of 45% of calories from fat (lard), 20% of calories from protein, and 35% of calories from carbohydrates in the previous study^38^. In contrast, our HFD consisted of 47.5% common diet, 20% sucrose, 20% fat (lard), 10% protein, 1% cholesterol and 0.5% sodium deoxycholate. Our study demonstrated that consistent with the amelioration of lipid and glucose disorders, low- or high-dose ASP significantly alleviated HFD-induced insulin resistance, indicating the hypoglycemic effect of ASP was related to the improvement of insulin resistance.

We then explored the molecular mechanism underlying the improvement of insulin resistance using western blot analysis. Insulin stimulates glycogen accumulation through a coordinated increase in glycogen synthesis and glucose transport. Insulin stimulation of IR/IRS/PI3K/Akt, which is the classic insulin signaling cascade, is required for GLUT translocation and the inhibition of kinases, such as GSK-3β^39^,^40^. Normally, the liver plays a key role in glucose metabolism, and impaired insulin signaling is pivotal for the development of insulin resistance. In the current study, western blot data revealed elevated expression levels of IR-β, phosphorylated IRS1, IRS1/2, phosphorylated PI3K, the p85 regulatory subunit of PI3K, phosphorylated Akt, Akt and membrane GLUT2, as well as decreased GSK-3β levels, in the livers of ASP-treated mice compared with the HFD-fed mice. These data suggest that ASP improves insulin resistance via the regulation IR/IRS/PI3K/Akt/GLUT/GSK-3β signaling proteins. Moreover, we examined the expression of phospho-JNK, which negatively regulates the insulin pathway and is abnormally elevated in the context of insulin resistance^41^. It has been demonstrated that the activation of JNK induces IRS-serine^307^ phosphorylation, which leads to a reduction in insulin-stimulated PI3K activity^42^. Our data showed that in addition to stimulating the IR/IRS/PI3K/Akt pathway, ASP also downregulates phospho-JNK expression in HFD-fed mice. This result indicates that the enhancement of the insulin pathway also contributes, at least in part, to the regulation of phospho-JNK in the liver.

HFD-induced insulin resistance is also associated with other physiological perturbations, such as altered levels of adipokines and inflammatory factors, as well as oxidative stress^33^. In this study, we examined serum TNF-α concentrations, but no variations were observed between the groups. However, ASP significantly reversed the reduced levels of serum adiponectin and resistin, which have been demonstrated to play key roles in the development of insulin resistance^43^. This result suggests that the ameliorative effects by ASP were associated with white adipose tissue. Importantly, as mentioned above, ASP significantly improved oxidative stress and the antioxidant system, demonstrating the key role of ASP in the amelioration of insulin resistance.

In summary, the present study demonstrated that chronic administration of the natural biomacromolecule ASP had appreciable therapeutic effects in HFD-induced fatty livers and hyperglycemia. Moreover, ASP treatment resulted in a considerable improvement in oxidative stress. The current study provides evidence that ASP could be employed an alternative dietary supplementation or health care product to ameliorate NAFLD and diabetic symptoms in populations that consistently consume HFDs.

## Methods

### Plant materials

The dry roots of *Angelica sinensis* (Oliv.) Diels (Umbelliferae) were obtained from Union Hospital and collected from Minxian (Gansu Province, China). Plant identification was performed by Professor Jinlan Ruan (Faculty of Pharmaceutical Science, Tongji Medical College of Huazhong University of Science and Technology, Wuhan, China), according to the identification standard of the Pharmacopoeia of the People’s Republic of China. The extraction and purification of the polysaccharide were performed as previously described^15^. The sugar content of ASP (MW 72.9 kD) was approximately 95.1%, and the constituent monosaccharides were arabinose, glucose and galactose, with a molar ratio of 1:2.5:7.5^44^.

### Animal experiments

The animal experiments were approved by *the Institutional Animal Care and Use Committee of Tongji Medical College, Huazhong University of Science and Technology*. The animal care and experimental procedures were carried out in accordance with *the Guidelines of the Institutional Animal Care and Use Committee of Tongji Medical College* and *the National Institutes of Health Guide for the Care and Use of Laboratory Animals.* Sixty male BALB/c mice (20 ± 2 g) were purchased from the Center for Experimental Animal Research (Wuhan, China) and maintained in polypropylene cages (six in each cage) in an air-conditioned room (25 ± 1 °C, relative humidity 50 ± 20%, 12-hour light/dark cycle). After a one-week acclimation period, the mice were started on regular chow or a HFD (47.5% common diet + 20% sucrose + 20% fat from lard + 10% protein + 1% cholesterol + 0.5% sodium deoxycholate). Metabolic dysfunction was induced by an 8-week treatment with a HFD, and the animals in this group were randomly allocated into the following four treatment groups of twelve mice each: one vehicle-treated metabolic dysfunction control group, and three ASP intervention groups (80, 160 and 320 mg·kg^−1^ per day, referred as LASP, MASP and HASP group, respectively). The ASP was dissolved in distilled water and administered orally by metallic gavage needle. Twelve mice were fed a STD, serving as a normal control group, and were orally administered distilled water (10 mL·kg^−1^ per day). All of the mice were allowed free access to normal pellet diet or a HFD during the experimental period.

### Monitoring of body weight and FBG with a glucose tolerance test and insulin response test, respectively

Body weight and glucose levels were measured from fasting mice at 7-day intervals. Blood for the FBG test was obtained from the tail vein of overnight fasting mice and examined using a one-touch glucometer (Bayer, German). The OGTT was performed one day before the mice were euthanized, in accordance with previously described methods^45^. Briefly, mice were deprived of food overnight and orally administered with a glucose solution at a dose of 3 g·kg^−1^. The blood samples were taken from tail tip and measured with a glucometer before and 30, 60, 90 and 120 min after glucose loading.

In addition, insulin resistance HepG2 cells were induced as previously described^46^ to perform the insulin response test after ASP treatment. Briefly, the cells were seeded into 6-well plates for 24 h and serum-starved for the following 24 h. After 24-h pretreatment with serum-free DMEM with normal (5.5 mM) or high (30 mM) concentration of glucose in the absence or presence of ASP, the response to insulin (100 nM for 10 min) was measured by detecting the expression of p-IRβ, p-IRS1, p-PI3K and p-Akt. Meanwhile, the PI3K inhibitor (LY294002, 20 μM) was added in the final 6 h as control groups.

### Measurement of biochemical parameters in the serum and liver

At the end of the study, blood was collected from the orbital sinus. The animals were then euthanized by cervical dislocation, and the liver tissues were harvested, weighed, and processed for subsequent analyses. Serum was collected after 3500 rpm centrifugation at 4 °C for 10 min. Liver tissue homogenates were prepared by high-speed stirring of liver tissue in a 10-fold volume (v/w) of ice-cold PBS or anhydrous alcohol (for estimation of lipids), followed by 12000 rpm centrifugation at 4 °C for 15 min. The supernatant was then collected for subsequent analysis.

TG, TC, LDL-C, HDL-C, ALT, AST, PK, HK, ROS, SOD, MDA, CAT, GSH and GPx levels in the sera or liver homogenates, as well as whole blood HbA1c, were determined using biochemical kits (NanJing JianCheng Bioengineering Institute, China) according to specifications. Insulin, TNF-α, hepcidin, adiponectin, resistin, G-6-pase and GK in the sera or liver homogenates were measured using commercially available ELISA kits.

### Histological assessment

The hepatic tissues were resected and fixed in 10% formalin solution and then embedded in paraffin using a tissue-embedding procedure. After fixation, the tissue sections were cut into 4-μm sections and stained with H&E. To assess hepatic lipid accumulation, liver samples were embedded in Tissue-Tek, and frozen sections were prepared for ORO staining using a routine method^47^. Photomicrographs were taken with a light microscope equipped with a camera (Olympus, Tokyo, Japan).

### Western blot analysis

For immunoblotting analysis, frozen liver samples were homogenized in RIPA lysis buffer (25 mM Tris-HCl, 25 mM NaCl, 0.5 mM EDTA, 1% Triton X-100 and 0.1% SDS) containing 1% protease inhibitor cocktail and 1 mM PMSF. The supernatant was collected after centrifugation (12000 × *g* at 4 °C for 15 min), and the total protein concentration was quantified using the BCA method, followed by mixing with loading buffer. The membrane protein was extracted according to the specifications of a commercial kit (Thermo Scientific Pierce). Equal amounts of proteins (100 μg) were separated by electrophoresis on 10 or 12% SDS polyacrylamide gels and transferred to nitrocellulose membranes (Millipore, Bedford, MA). Membranes were blocked for 2 h at room temperature with 5% skimmed milk in TBST. The membranes were then incubated with primary antibodies against p-InRβ (Abcam 62321), InRβ (CST#3025), p-IRS1 (Abcam 46800), IRS-1 (CST#3407), IRS-2 (CST#4502), p-PI3K (CST#4228), PI3K (CST#4257), p-Akt (CST#4060), Akt (CST#4685), p-JNK (CST#9255), SIRT1 (CST#9475), p-AMPK (CST#2535), AMPK-α (Abcam 133278), PPARγ (Abcam 191407), GLUT2 (Abcam 54460) and GSK-3β (Santa Cruz Biotechnology sc-377213) at a dilution ratio of 1:1000. The membranes were washed three times and incubated for 1 h at room temperature in TBST with anti-rabbit or anti-mouse HRP-conjugated secondary antibodies at a dilution ratio of 1:4000. The membranes were then treated with enhanced chemiluminescence (ECL, Thermo Scientific). The blot intensities were evaluated by densitometric analysis using ImageJ software and standardized by expressing the density of each band of interest relative to that of beta-actin, which severed as a reference.

### Statistical analysis

All of the data are expressed as the mean ± S.D. The data were assessed by SPSS version 19.0 software (SPSS, Chicago, IL, USA). A non-parametrical Kolmogorov-Smirnov test was employed for verifying a normal distribution of the data. Differences between groups were assessed by one-way analysis of variance (ANOVA) with either LSD (assuming equal variances) or Dunnett’s T3 (not assuming equal variances) for post-hoc analyses. Statistical significance was considered at *P* < 0.05. The HOMA-IR index was calculated as follows: HOMA-IR = FBG (mmol·L^−1^) × Insulin (mU·L^−1^)/22.5.

## Additional Information

**How to cite this article**: Wang, K. *et al.* Chronic administration of *Angelica sinensis* polysaccharide effectively improves fatty liver and glucose homeostasis in high-fat diet-fed mice. *Sci. Rep.* **6**, 26229; doi: 10.1038/srep26229 (2016).

## Supplementary Material

Supplementary Information

## Figures and Tables

**Table 1:** Metabolic parameters of HFD-induced mice^a^.

	STD	HFD	LASP	MASP	HASP
Glucose (mmol·L^−1^)	3.0 ± 0.4	5.6 ± 0.8^##^	3.7 ± 0.8^**^	3.6 ± 0.6^**^	3.3 ± 0.5^**^
Hepatic glycogen (mg·g^−1^)	17.71 ± 5.93	4.87 ± 2.41^##^	14.59 ± 2.48^**^	12.63 ± 2.55^**^	15.16 ± 3.67^**^
HbA1c (OD value)	41.32 ± 7.52	63.41 ± 5.90^##^	46.52 ± 3.44^**^	47.62 ± 14.34^**^	45.28 ± 8.85^**^
Insulin (mIU·L^−1^)	29.10 ± 3.32	25.27 ± 8.95	26.67 ± 4.76	33.08 ± 3.29^*^	27.86 ± 2.14
HOMA-IR	3.89 ± 0.71	6.16 ± 1.92^##^	4.21 ± 1.00^*^	5.37 ± 0.47	4.11 ± 0.80^**^
Serum TG (mmol·L^−1^)	0.76 ± 0.13	0.99 ± 0.16^##^	1.06 ± 0.11	0.80 ± 0.12^*^	0.60 ± 0.15^**^
Serum TC (mmol·L^−1^)	3.13 ± 0.59	4.78 ± 0.93^##^	3.72 ± 0.64^**^	3.38 ± 0.64^**^	3.10 ± 0.51^**^
Serum HDL-C (mmol·L^−1^)	1.12 ± 0.16	0.80 ± 0.15^##^	0.75 ± 0.11	0.87 ± 0.17	1.12 ± 0.23^**^
Serum LDL-C (mmol·L^−1^)	2.28 ± 0.63	3.91 ± 0.92^##^	2.89 ± 0.66^*^	2.61 ± 0.67^**^	2.34 ± 0.50^**^
Serum resistin (μg·L^−1^)	13.43 ± 0.55	11.96 ± 1.01^#^	11.98 ± 0.69	12.67 ± 1.00	13.17 ± 0.60^*^
Serum adiponectin (μg·L^−1^)	141.03 ± 3.62	114.15 ± 5.66^##^	137.34 ± 5.60^**^	124.27 ± 2.17	125.38 ± 6.25
Serum hepcidin (μg·L^−1^)	17.50 ± 0.44	19.07 ± 0.66	18.08 ± 0.43	17.03 ± 0.20	18.22 ± 0.52
Serum TNF-α (pg·mL^−1^)	754.74 ± 19.55	730.46 ± 12.12	769.86 ± 17.45	810.61 ± 13.67	754.44 ± 23.25

^a^The values are given as the mean ± S.D. (n = 12). ^#^*P* < 0.05, ^##^*P* < 0.01 versus the STD group. *P < 0.05, **P < 0.01 versus the HFD group.
