# Supplementary material for: Chronic administration of Angelica sinensis polysaccharide effectively improves fatty liver and glucose homeostasis in high-fat diet-fed mice
Source: Sci Rep. 2016 May 18;6:26229. doi: 10.1038/srep26229 (PMC4870572; doi:10.1038/srep26229)
Supplement: Supplementary Information [file srep26229-s1.pdf]

Supplementary Information

Chronic administration of *Angelica sinensis* polysaccharide effectively improves fatty liver and glucose homeostasis in high-fat diet-fed mice

Kaiping Wang[1], Peng Cao[1], Hanxiang Wang[1], Zhuohong Tang[1], Na Wang[3], Jinglin Wang[2], Yu Zhang[2,*]

[1]Hubei Key Laboratory of Natural Medicinal Chemistry and Resource Evaluation, Tongji Medical College of Huazhong University of Science and Technology, 430030, Wuhan, China

[2]Union Hospital of Huazhong University of Science and Technology, Department of Pharmacy, No. 1227, Jiefang Road, 430030, Wuhan, China

[3]Department of Pharmacy, Renmin Hospital of Wuhan University, No. 99, Zhangzhidong Road, 430060, Wuhan, China

[*]Corresponding author. E-mail: zhangwkp@163.com; Fax: +86 27 63559222; Tel: +86 27 63559222

PPARγ

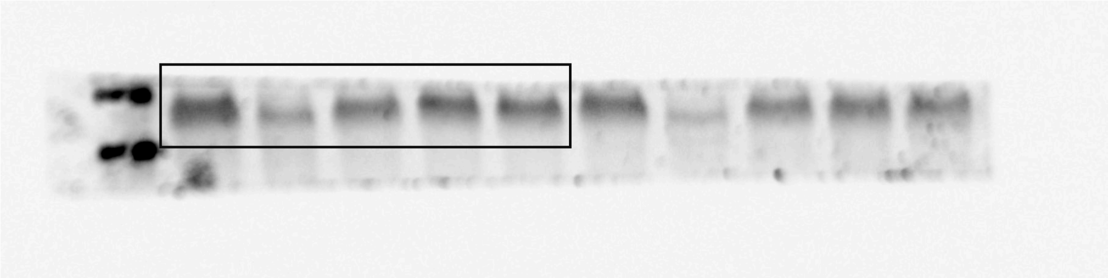

SIRT1

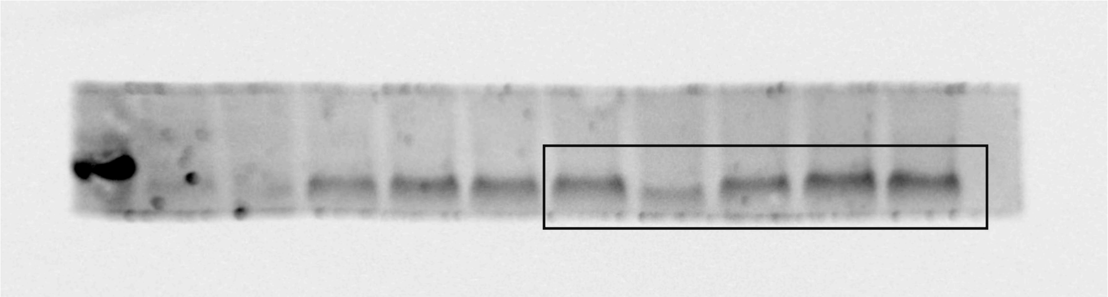

p-AMPK

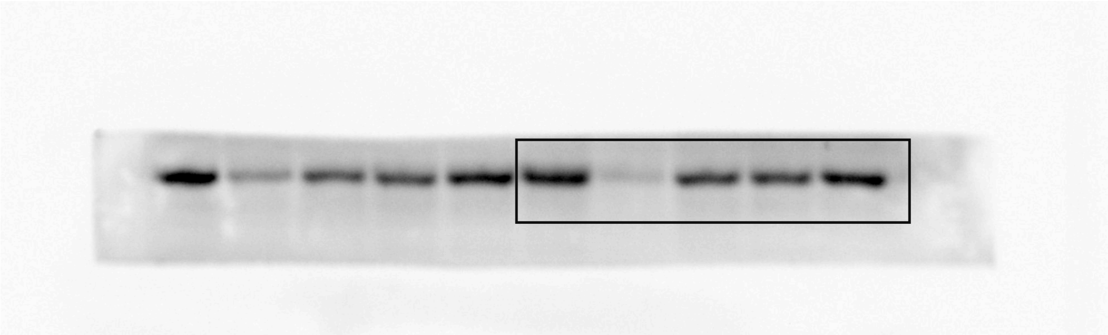

AMPK

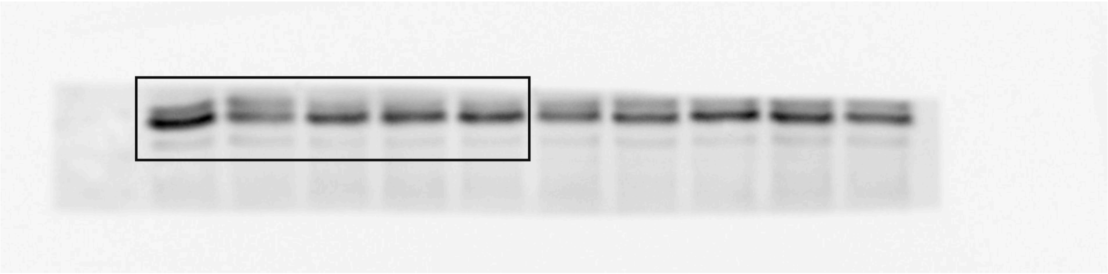

β-actin

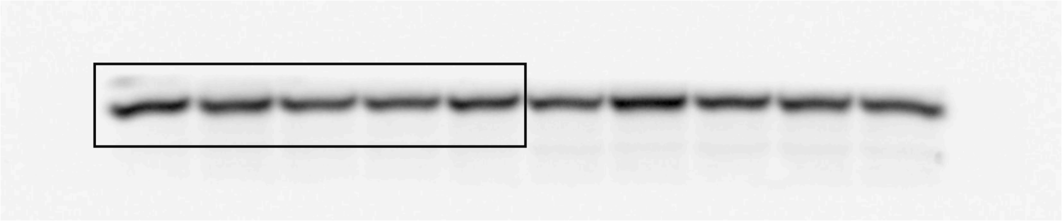

Figure S1. Presentation of original immunoblot shown in Figure 2C.

The cropped parts of immunoblot were indicated with black boxes.

p-IR

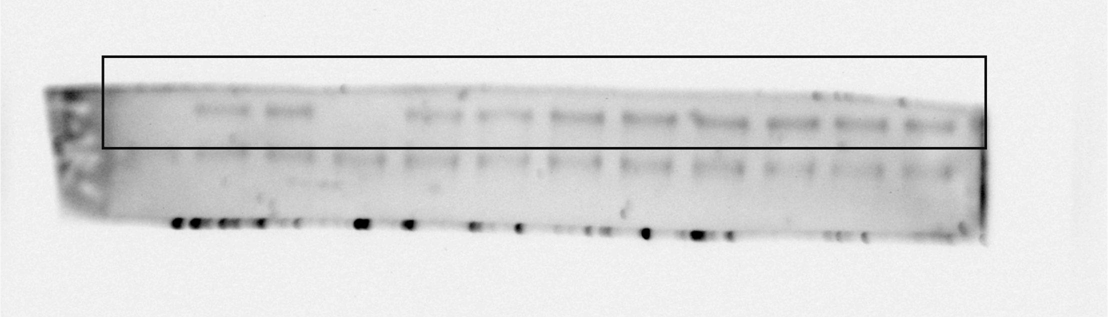

p-IRS1

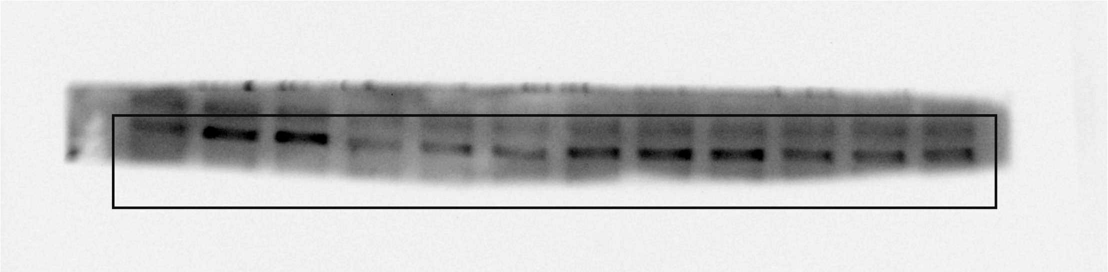

p-PI3K

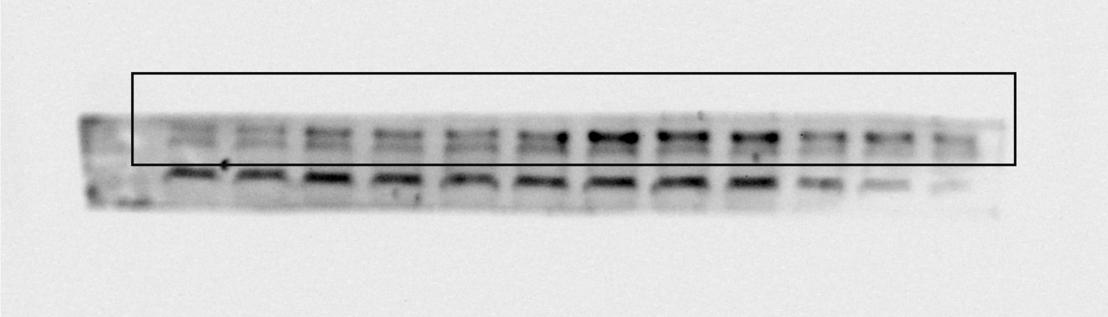

p-Akt

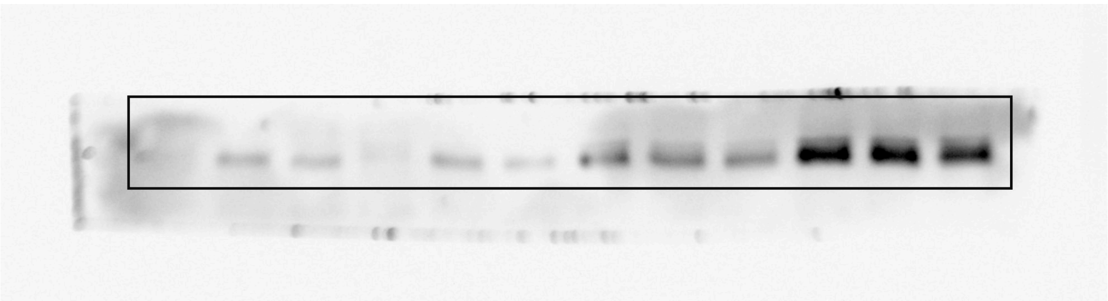

GAPDH

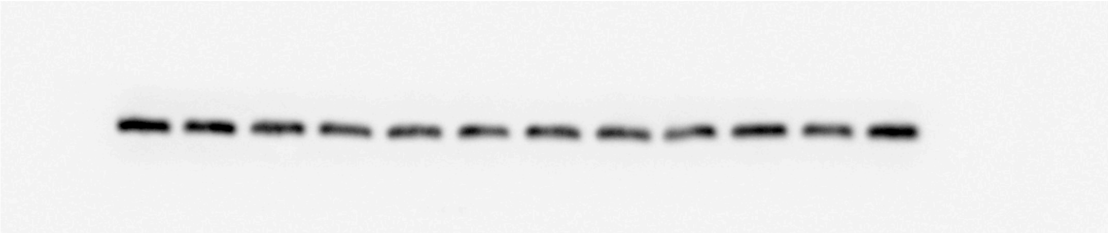

Figure S2. Presentation of original immunoblot shown in Figure 4C.

The cropped parts of immunoblot were indicated with black boxes.

IR-β

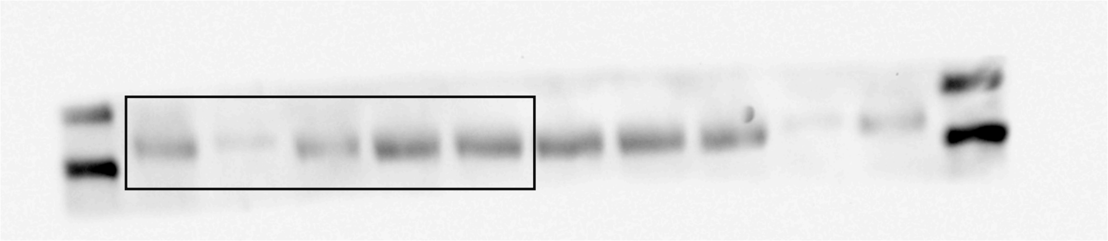

p-IRS1

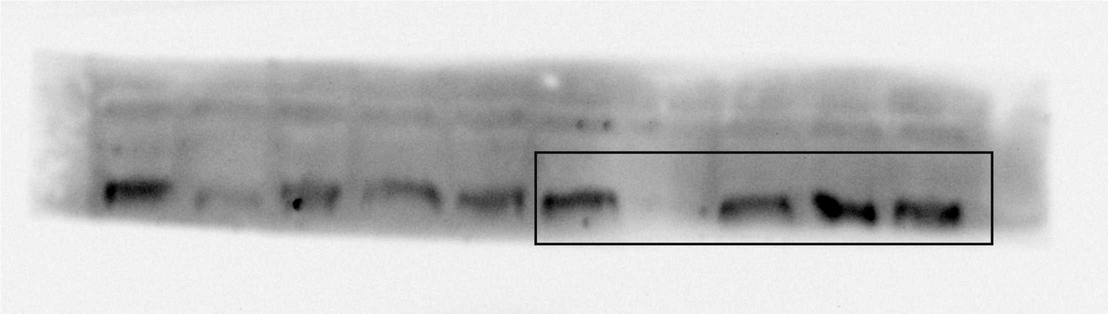

IRS1

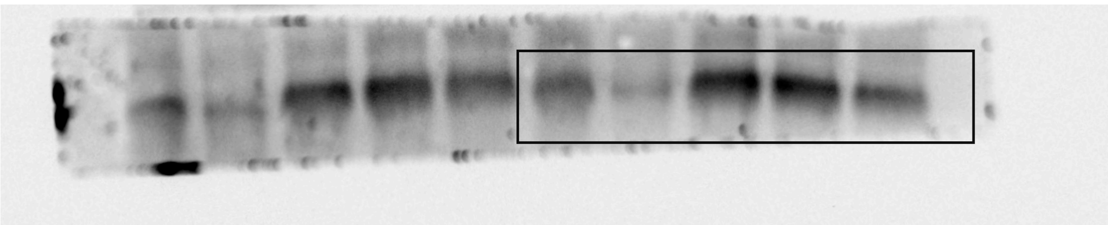

IRS2

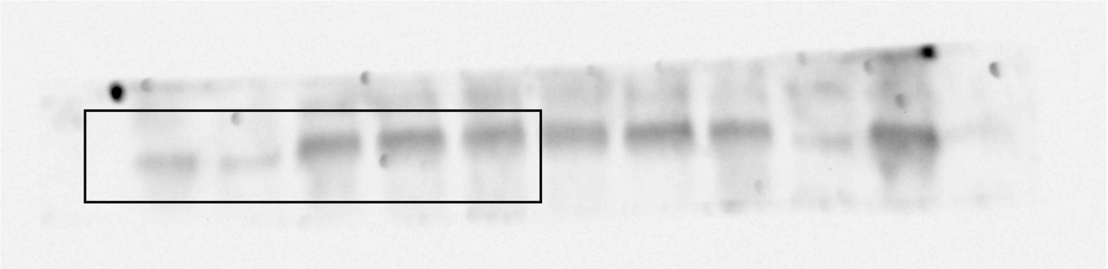

p-PI3K

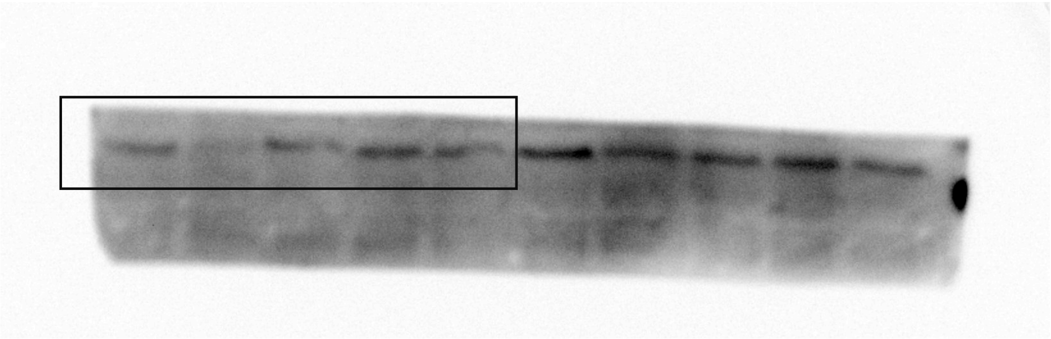

PI3K

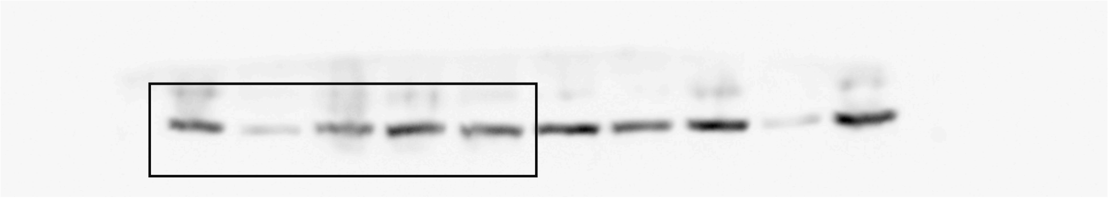

p-Akt

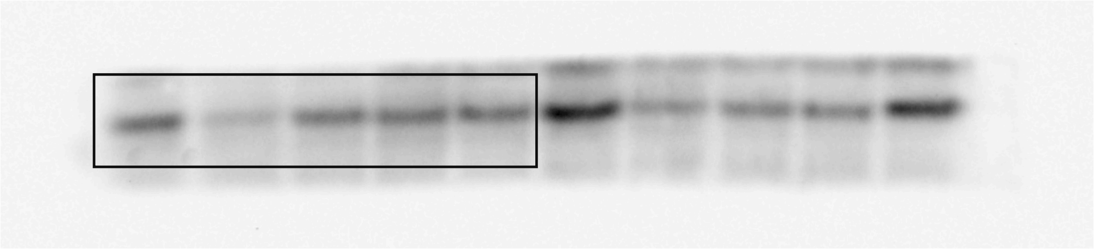

Akt

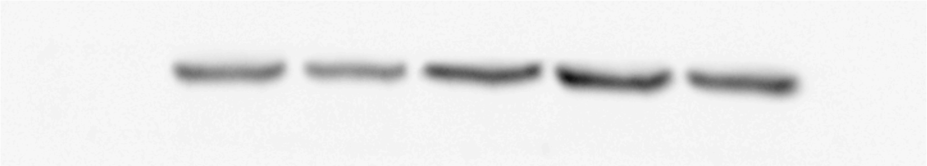

total-GLUT2

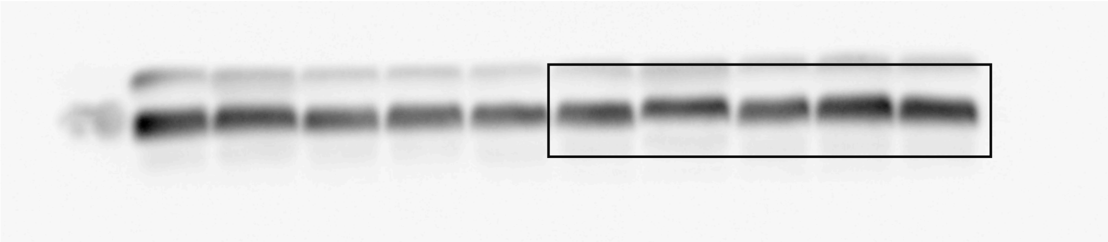

membrane-GLUT2

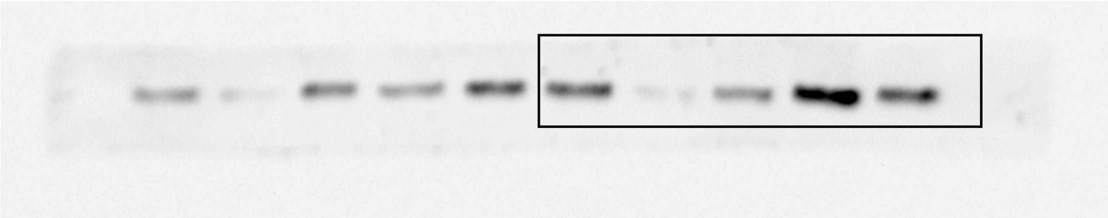

GSK-3β

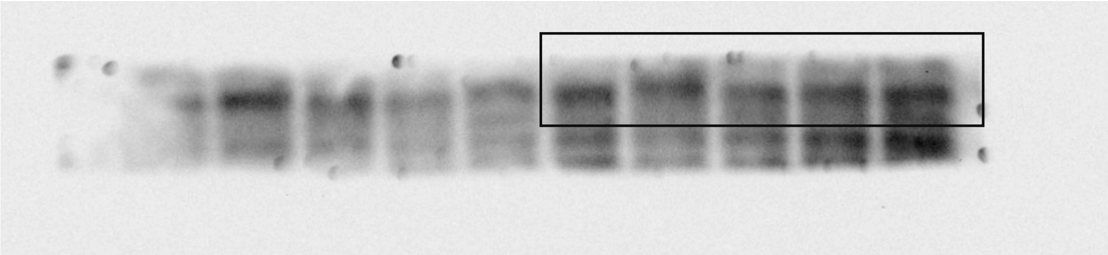

p-JNK

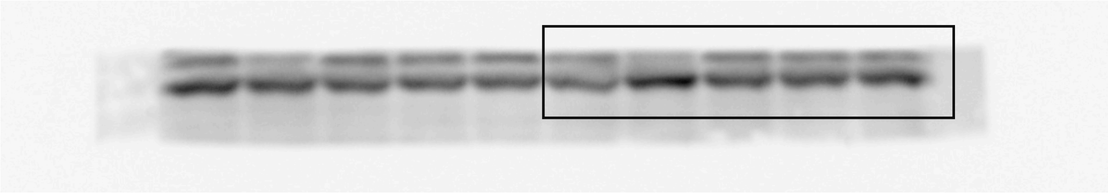

β-actin

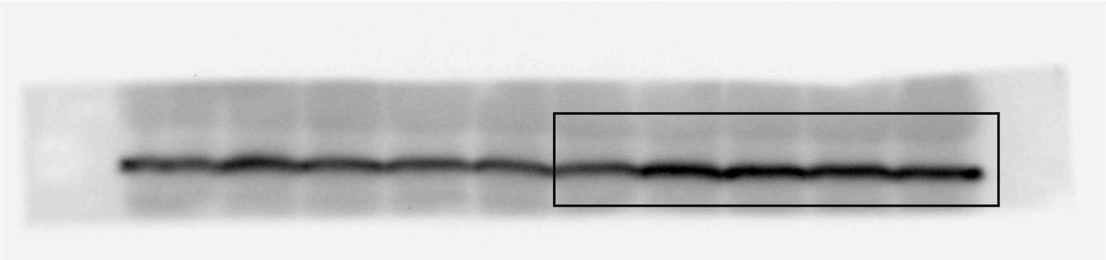

Figure S3. Presentation of original immunoblot shown in Figure 6.

The cropped parts of immunoblot were indicated with black boxes.
